# Pleuro-Pneumonia

**Published:** 1844-09-21

**Authors:** 


### PLEURO-PNEUMONIA.

F. C., ætat. 28, admitted into Guy's Hospital, Oct. 11th, 1843, under the care of Dr. Barlow. A muscular and hitherto healthy man, who has been employed as a porter in a tobacco warehouse, and also at the London Docks. Four days ago, when much heated, he was exposed to the rain for some time. He felt no inconvenience on that day, and went to bed at night in his usual health, but on the following morning he was too ill to leave his bed, complaining of rigors, headach, sickness, and some vomiting.

*Present Symptoms.*—There is a hard, frequent, and short cough, attended with much pain over the sternum, and passing through to between the scapulæ; this pain is much aggravated by a deep inspiration. There is copious expectoration, of a rusty color, but which is more frothy than viscid. There is also pain, on pressure, over the region of the liver, and the conjunctivæ are tinged of a yellow colour. He complains of occasional acute pains in the right shoulder. The right side of the chest is dull on percussion, inferiorly, and there is a dry crepitation over the middle lobe of the lung on that side, in which situation there is also œgophony. The skin is pungently hot and dry, excepting some moisture at the palms of the hands. There is also a yellow tinge over the surface. Tongue white and furred; pulse quick and full, but easily compressible; urine high coloured. Ordered cupping on the right side of the chest to ten ounces.

℞ Protochloride of mercury, two grains; opium, a quarter of a grain. Mix for a pill every four hours.

12. Cough continues. The sputa are of a more yellow colour, and the patient remarks that they are bitter to the taste. Bowels open once since admission; fæces dark coloured; tongue rather cleaner; pain on pressure over the liver continues.

℞ Antimonial wine, twenty minims; sulphate of magnesia, a drachm; tincture of orange-peel, a drachm; distilled water, an ounce and a half. Mix for a draught every six hours. Repeat the pills.

13. Cough, &c. much the same; bowels much relaxed. The dose of antimonial wine was increased to half a drachm.

℞ Protochloride of mercury, one grain: opium half a grain. Mix for a dose every four hours.

14. Did not sleep at all last night from continued lancinating pain in the right side. This moring he is somewhat easier. There is now a distinct, dry rubbing of the pleura to be heard over the middle lobe of the lung, the crepitation over that part having ceased. Posteriorly there is a loss of respiratory murmur and well-marked bronchophony. Percussion elicits general dulness on the right side, but this is most evident posteriorly. He is unable to take a full inspiration. Sputa slightly tenacious. Cupping on the right side of the chest to eight ounces. The mixture repeated. A pill to be taken every six instead of every four hours.

15. Passed a better night, the acute pain of the right side having left him; he still, however, complains of general soreness of that side. The rubbing sound is still distinctly audible over the same region as before. Bronchopony posteriorly is somewhat œgophonic. Two loose evacuations since yesterday morning.

17. Cough much diminished; complains but little of his chest, except of slight dyspnœa; sputa frothy, but tenacious, and more free from colour. Ordered compound ipecacuanha-powder, ten grains, every night. The mixture repeated. The calomel and opium pill discontinued.

31. He has been improving, and coughs but little; no expectoration; tongue clean; skin moist; pulse fuller, but of moderate frequency. There is still dulness posteriorly on the right side of the chest; inferiorly bronchophony.

Nov. 2. Ordered conium mixture.

6. Presented cured.

*Remarks by Dr. Barlow.*

Although in this case we have little more than an instance of pleuro-pnuemonia, with some degree of bronchitis—a form of disease of very frequent occurrence in this climate, and one which, when detected sufficiently early, and subjected to prompt and decided, but not violent treatment, we can generally hope to combat successfully—still there are certain points in its history which are capable of affording illustration of the diagnosis and treatment of pneumonia, the former of which is the more important, since, if the disease be overlooked in its commencement, a fatal result or permanent injury to the health and comfort of the patient may ensue.

At the time of his admission, the more obvious symptoms presented by this man were not manifestly those which are most generally regarded as indicative of pneu[illegible]onia. There was an oppressed and almost apathetic expression in his countenance, which, at first sight, seemed to belong more to continued fever than to thoracic inflammation; the pulse again, though quick and full, was compressible, and the cough and expectoration were rather those of bronchitis, a very frequent complication of contiued fever, than of pneumonia. There was, however, one symptom which, although it is not exclusively pathognomonic of pneumonia, is, nevertheless, so constantly associated with it, especially in its earlier stages, that its presence ought always to excite suspicion of the lungs being inflamed, and to suggest the expediency of making a most careful examination of the chest. I need hardly say that I allude to the dry pungent heat of the skin, which was noticed on the patient's first admission. The value of this symptom in relation to pneumonia, has been so often pointed out in this hospital by Dr. Addison, that it is here needless to insist upon it. It may, however, be well to observe, that its great value consists in its presenting itself so readily to the sense of touch upon the first examination of a patient, and that although it will, no doubt, sometimes mislead if relied upon singly (as will every symptom which has been laid down as exclusively pathognomonic in any disease whatever), still, when viewed in its proper light as one which ought to direct especial attention to the condition of the lungs, it is, I believe, among the most valuable additions which have been made in late years to our means of diagnosis. The result was that auscultation confirmed the opinion which had been suggested by the condition of the skin. For it was found that the resonance of the chest was somewhat diminished in the situation of the middle lobe of the right lung, where a dry crepitation was heard, indicative of the first stage of pneumonia. The dulness on percussion, indeed, extended downwards over the lower lobe, where the sound of the voice was œgophonic, indicating that the pleura covering the inflamed lung participated, as is frequently the case in the inflammation, and that some effusion had ensued. It is at all times most satisfactory, when the diagnosis formed by a topical examination of the organ suspected, and that derived from the constitutional symptoms, are thus mutually confirmatory of each other.

There was another symptom present in this instance, which deserves consideration in connection with pneumonia, I mean the icteric tint of the surface. This appearauce has been sometimes supposed to depend upon the increased determination of the blood to superficial vessels, which commonly takes place in pneumonia; but I think that if this explanation were correct, we ought to find it in all cases of pneumonia, in which there is, as almost constantly happens, great heat of surface; whereas this icteric tint, though frequently present under such circumstances, is frequently absent. The evidence of the presence of bile in the circulation, afforded both by the urine and the sputa, tends to show that the colour of the skin was really that of true jaundice; and that such a disease should often co-exist with pneumonia (especially of the right lung,) is what we have been inclined to anticipate, from the near relation as regards position, circulation, and function, which exists between the liver and the lungs.

The principles upon which the patient was treated, are, perhaps, sufficiently apparent from the history. Local was preferred to general blood-letting, both because the powers of the patient seemed hardly to justify the latter, and because I believe, that unless it be imperatively called for by the condition of the patient, blood-letting is, as has been observed by Dr. Stokes, less effective than cupping in subduing the local affection of the lungs.

The use of calomel, antimony, and opium, is a practice which has been sanctioned by long experience in the hospital, as well in pneumonia complicated with pleuratic inflammation, as in the more simple form of the disease; and although the treatment of antimony alone has been highly extolled by several French practitioners, I am, nevertheless, much disposed to concur in the opinion expressed by Dr. Stokes, that although it is a very effective medicine in inflammation of the mucous membrane of the air passages, it is not so serviceable as calomel in inflammation of the lungs themselves.—*Lon. Lancet.*

